# Light-emitting diodes and their potential in callus growth, plantlet development and saponin accumulation during somatic embryogenesis of *Panax vietnamensis* Ha *et* Grushv.

**DOI:** 10.1080/13102818.2014.1000210

**Published:** 2015-01-14

**Authors:** Duong Tan Nhut, Nguyen Phuc Huy, Ngo Thanh Tai, Nguyen Ba Nam, Vu Quoc Luan, Vu Thi Hien, Hoang Thanh Tung, Bui The Vinh, Tran Cong Luan

**Affiliations:** ^a^ Tay Nguyen Institute for Scientific Research, Vietnam Academy of Science and Technology, Vietnam; ^b^ Research Center of Ginseng and Medicinal Materials, National Institute of Medicinal Materials, Vietnam

**Keywords:** callus, LEDs, *Panax vietnamensis* Ha *et* Grushv., spectrum

## Abstract

In recent years, LED (light-emitting diode) has been the subject of research within the field of plant growth and development. However, there has been little discussion about using LED *in vitro* cultures of *Panax vietnamensis*, one of the important medicinal plants belonging to the *Panax* genus. This study examines the influence of various LED lamps on callus growth and plant formation of *P. vietnamensis*. Results show significant differences in growth and development, as various light conditions were suitable for different stages. Callus of 70 mg in fresh weight cultured under yellow LEDs resulted in growth of 1197 mg in fresh weight and 91.7 mg of dry weight, within a period of three months. The most effective plant formation was obtained when embryogenic calli were cultured under the combination of 60% red LED and 40% blue LED with an average of 11.21 plantlets per explant; the shoot clump fresh weight and dry weight were of 1147 and 127 mg, respectively, and the average plant height was 3.1 cm. It was also shown that this light condition was the most efficient for *P. vietnamensis in vitro* plant growth and development. This study provided additional evidence regarding the influence of different LEDs on ginsenoside production applying high-performance liquid chromatography (HPLC) analysis with photo-diode array (PDA) detection at ultraviolet (UV) wavelength 203 nm. The highest MR_2_ content was recorded when plants maintained under 20% red LED combined with 80% blue LED. However, the highest Rg_1_ and Rb_1_ content was found under fluorescent light. The results presented might provide new strategies using LEDs for adequate micropropagation protocols of *P. vietnamensis*.

## Introduction

*Panax vietnamensis* Ha *et* Grushv. belong to the Araliaceae family and it is one of the most precious ginsengs. Researchers show an increased interest in this plant due to its high saponin content, especially the dammaran group, including MR_2_, Rg_1_ and Rb_1_.[1] Most of the previous studies have only focused on saponin content analysis and pharmacology effects. Nhut et al*.* [2] investigated different media for callus, shoot and adventitious root biomass proliferation, which primarily quantified the saponin content of *P. vietnamensis in vitro* biomass.[2]

Light irradiation has remarkable effects on plant cell and tissue growth and secondary metabolite biosynthesis. A considerable amount of information on light-emitting diode (LED) has been extensively described in literature, as a novel lighting source in plant tissue culture growth with several advantages such as small size, low mass, a long functional life, narrow spectral output, etc. compared with the traditional fluorescent lamps.[3]

However, there is little investigation on *P. vietnamensis* cultures using LEDs, as there are no studies covering the utilisation of yellow, green and white LEDs in *P. vietnamensis* cultures. The purpose of this work is to examine the influence of various kinds of LED (blue, green, yellow, red and white LEDs and red LED in combination with blue LED at different ratios) in order to define the effective lighting conditions for biomass productivity and saponin accumulation*.* 3U compact fluorescent lamps, fluorescent lamps and darkness were used as the control on callus growth and plant formation of *P. vietnamensis.* The aim of this study is to provide a new insight in *P. vietnamensis* culturing.

## Materials and methods

### Materials and culture media

Clusters of 70 mg callus derived from leaf segments of *P. vietnamensis* that were cultured on Schenk and Hildebrandt (SH) medium [4] containing 0.2 mg/L thidiazuron, 1.0 mg/L 2,4-D (2,4-dichlorophenoxyacetic acid), 30 g/L sucrose and 9 g/L agar for callus proliferation.[5]

Calli were obtained and transferred into Murashige and Skoog medium [6] supplemented with 1 mg/L 2,4-D, 0.2 mg/L kinetin, 0.5 mg/l NAA (*α*-naphthaleneacetic acid), 30 g/L sucrose and 8.5 g/L agar in order to develop embryogenic calli.

Clusters of 30 mg embryogenic callus were then cultured on SH medium with 1 mg/L BA (6-benzyladenine), 0.5 mg/L NAA, 30 g/L sucrose and 9 g/L agar to attain plantlets.[5]

Following this, two centimetre plantlets were selected and placed on SH medium supplemented with 0.5 mg/L BA, 0.5 mg/L NAA, 30 g/L sucrose and 9 g/L agar [7] in order to estimate further growth and development. pH was adjusted to 5.7–5.8 prior to autoclaving at 121 °C, 1 atm for 30 min using SA-600 Sturdy Autoclave (Sturdy Industrial Co., LTD., Taiwan).

### Lighting conditions

Cultures were maintained in darkness and under 16 different lighting conditions including (1) 3U compact fluorescent lamps, (2) fluorescent lamps, (3) blue, (4) green, (5) yellow, (6) red and (7) white LEDs, and red LED in combination with blue LED at different ratios including (8) 90:10, (9) 80:20, (10) 70:30, (11) 60:40, (12) 50:50, (13) 40:60, (14) 30:70, (15) 20:80 and (16) 10:90 at the light intensity of 20–25 µmol m^−2^ s^−1^, temperature of 25 ± 2 °C and relative humidity of 55%–60%.

### Qualitative and quantitative saponin analysis

*In vitro P. vietnamensis* plants were used for saponin analysis. The procedures for saponin extraction, HPLC and thin layer chromatography (TLC) analysis were previously described by Zhai et al. [8] and Odani and co-workers.[9,10]

Plantlets were collected after 12 weeks of culture. The samples were cleaned, dried at 60 °C, ground (at powder grade) and stored at room temperature until utilisation. Reference samples of *P. vietnamensis* and standard compound MR_2_ were supported by Research Center of Ginseng and Medicinal Materials. Ginsenoside-Rb_1_ (Rb_1_) and ginsenoside-Rg_1_ (Rg_1_) were purchased from Wako Pure Chemical Industries, Ltd., Japan.

HPLC system: Supelco RP C18 column (250 mm × 4.6 mm; I.D. 5 mm) and a SPD-M20A-PDA detector (Shimadzu) were used. HPLC parameters: volume injection of 20 mL; flow rate of 0.5 mL/min. Column temperature was kept at 25 °C.

Sample (0.5 g) was exhaustively extracted in methanol using a sonicator (10 mL methanol × 6 times). The extracts were joined together and concentrated by an evaporator to dry residues. The residues were dissolved in 20 mL of water and fractionated with ether ethylic and *n*-butanol, respectively. The ether ethylic fraction was discarded and the *n*-butanol was collected and evaporated under vacuum pressure in order to yield the dried extract. The resulted dried extract was continuously dissolved with a mixture of acetonitrile water solvent (2:1, v/v) and a volume of 5 mL was filtered through a 0.45 μm membrane. The filtrate was finally injected in the HPLC system for quantitative determination of saponins using the calibration curve method.

### Data collection and analysis

All treatments were in triplicates and each replicate with 10 culture vessels. Data were scored after 12 weeks of culturing and analysis of variance was performed. The means were compared using Duncan's multiple range Test using SPSS (Version 16.0) at *P* value = 0.05.[11]

## Results and discussion

### Callus proliferation

The impact of light on higher plants mainly occurs in two aspects – to provide the energy source required by the plant and to be a signal received by a photoreceptor to regulate the growth, differentiation and metabolism.[12] The results of this study indicated that yellow LED light with the wavelength of 570–590 nm was effective for callus growth of *P. vietnamensis* with significantly higher values of callus fresh and dry weight compared to those treated with fluorescent lamp and other light sources. In 1996, Soni and Swarnkar published a study showing that blue and yellow spectra evoked callus and shoot bud formation from leaf cultures of *Vigna aconitifolia*.[13] Ouyang et al. [14] also demonstrated that light intensity and the spectral quality had an effect on *Cistanche deserticola* callus culture and the biosynthesis of phenylethanoid glycosides.[14] Light plays an important role in regulating the growth, differentiation and metabolism. Furthermore, higher plants cultured *in vivo* had at least three types of photoreceptors that selectively absorbed different spectral light.[14]

**Figure 1.**
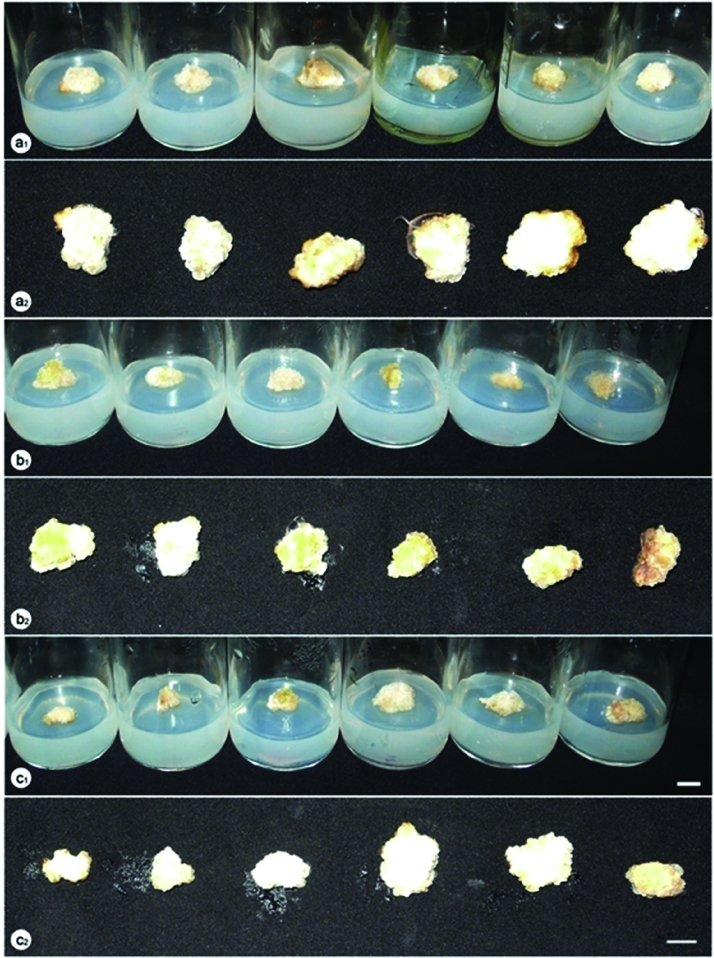
Callus proliferation under different lighting conditions after 12 weeks of culture. a_1_, a_2_: fluorescent lamp, 3U compact fluorescent lamp, white LED, darkness, green LED, yellow LED (from left to right); b_1_, b_2_: blue LED, red LED combined with blue LED at the ratios of 10:90, 20:80, 30:70, 40:60 and 50:50 (from left to right); c_1_, c_2_: red LED, red LED combined with blue LED at the ratios of 90:10, 80:20, 70:30, 60:40 and 50:50 (from left to right).

Significant differences in callus growth were observed among the explants cultured under different lighting conditions (Table 1 and Figure 1). Yellow LED was observed to be the best treatment for callus growth with highest fresh and dry weight at 1197 and 91.7 mg, respectively. This is the very first study on *P. vietnamensis* clarifying the effect of yellow, green and white LED on callus growth, and yellow LED was found to promote this process. Following the treatment with yellow LED, a considerable improvement in the growth of callus was recorded when the callus clusters were maintained under 60% red LED combined with 40% blue LED compared to those cultured under fluorescent lamps. There were no significant differences between the callus growth under 3U compact fluorescent lamp, green and white LED, combination of red LED and blue LED at the ratios of 70:30 and 50:50, the darkness and fluorescent lamp. Red and blue LEDs, and the combination of red LED and blue LED at the ratios of 90:10, 80:20, 40:60, 30:70, 20:80 and 10:90 were found to inhibit the proliferation of the callus. Among these treatments, the minimum of callus fresh and dry weight were scored under red LED.

**Table 1.** Influence of different lighting conditions on callus growth of *P. vietnamensis* after 12 weeks of culture.

Treatment	Fresh weight (mg)	Dry weight (mg)
Fluorescence	763^cd*^	66.1^c^
3U	640^de^	46.6^fg^
Darkness	771^bc^	64.5^c^
Yellow	1197^a^	91.7^a^
Green	823^bc^	63.3^cd^
White	606^de^	56.6^de^
Red	287^g^	21.6^j^
90 red:10 blue	370^fg^	28.4^ij^
80 red:20 blue	602^de^	40.5^gh^
70 red:30 blue	888^b^	69.3^bc^
60 red:40 blue	930^b^	79.0^b^
50 red:50 blue	730^cd^	67.3^bc^
40 red:60 blue	525^ef^	51.7^ef^
30 red:70 blue	510^ef^	41.8^gh^
20 red:80 blue	480^ef^	39.0^gh^
10 red:90 blue	422^fg^	33.7^hi^
Blue	370^fg^	33.5^hi^

Note: Different letters (*) in the same column indicate significantly different means using Duncan's test at *P* = 0.05.

### Plant formation

**Figure 2.**
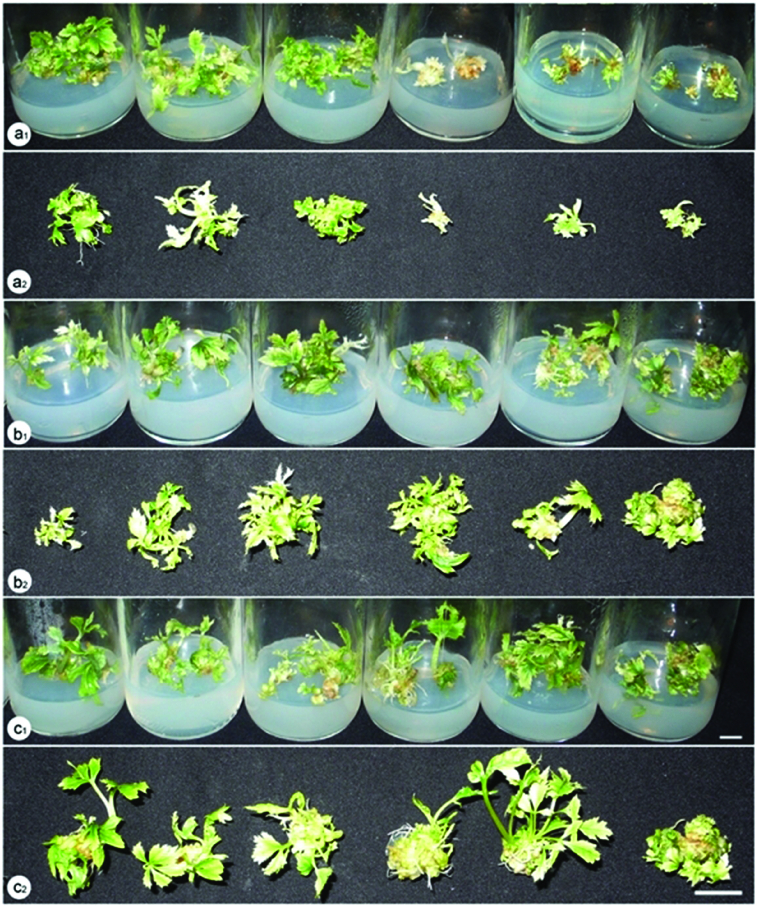
Plant formation from embryogenic callus of *P. vietnamensis* under different lighting conditions after 12 weeks of culture. a_1_, a_2_: fluorescent lamp, 3U compact fluorescent lamp, white LED, darkness, green LED, yellow LED (from left to right); b_1_, b_2_: blue LED, red LED combined with blue LED at the ratios of 10:90, 20:80, 30:70, 40:60 and 50:50 (from left to right); c_1_, c_2_: red LED, red LED combined with blue LED at the ratios of 90:10, 80:20, 70:30, 60:40 and 50:50 (from left to right).

Another interesting observation was that the type of light source also affected the plant formation of *P. vietnamensis* from embryogenic callus cultured *in vitro* (Table 2 and Figure 2). It can be seen from the data in Table 2 that the most effective treatment in plant formation was succeeded when embryogenic calli were placed under 60% red LED plus 40% blue LED after 12 weeks of culture, producing the highest values of fresh and dry weight, average plant height and number of plants per explant 1147 and 127 mg, 3.1 cm and 11.21 plants, respectively. The plant formation ability under this lighting condition was much higher than that under traditional lighting source for plant cell, tissue and organ culture with the fresh and dry weight of 505 and 49 mg, average plant height of 1.88 cm and 5.83 plants per explant. Statistical analysis also revealed that the combination of red and blue LEDs at ratios of 80:20, 70:30 and 50:50 were also significantly positive for the plant formation whilst no difference under other combinations of these LEDs (90:10, 40:60, 30:70 and 20:80) were found. The darkness was detected as an unsuitable condition for plant formation from somatic embryos of this crop with very low values of fresh and dry weight, average plant height and number of plants per explant (Table 2). Furthermore, when embryogenic clusters were cultured in darkness, there was a lack of chlorophyll in the plants (Figure 2). There was no increase of plant formation associated with the utilisation of yellow, green, white, red and blue LEDs (Table 2).

**Table 2.** Influence of different lighting conditions on plant formation of *P. vietnamensis* after 12 weeks of culture.

Treatment	Fresh weight (mg)	Dry weight (mg)	Average height (cm)	No. of plants/explant
Fluorescence	505^de*^	49^cd^	1.88^c^	5.83^de^
3U	546^de^	51^cd^	1.78^c^	6.503^d^
Darkness	206^j^	20^h^	1.10^fg^	3.67^h^
Yellow	244^ij^	27^gh^	0.91^g^	4.43^fgh^
Green	240^j^	25^gh^	1.15^ef^	4.17^gh^
White	320^hi^	33^fg^	1.52^d^	5.50^ef^
Red	368^gh^	34^fg^	1.35^de^	4.83^ef^
90 red:10 blue	565^cd^	55^cd^	1.50^d^	5.00^ef^
80 red:20 blue	673^bc^	60^c^	1.92^c^	7.67^c^
70 red:30 blue	778^b^	74^b^	2.43^b^	9.50^b^
60 red:40 blue	1147^a^	127^a^	3.10^a^	11.21^a^
50 red:50 blue	598^cd^	59^c^	1.98^b^	7.67^c^
40 red:60 blue	430^ef^	45^de^	1.94^b^	6.33^d^
30 red:70 blue	422^ef^	43^de^	1.80^c^	6.20^d^
20 red:80 blue	421^ef^	38^ef^	1.38^d^	5.83^de^
10 red:90 blue	380^fg^	38^ef^	1.32^de^	5.00^ef^
Blue	288^hi^	28^gh^	1.15^ef^	4.50^fgh^

Note: Different letters (*) in the same column indicate significantly different means using Duncan's test at *P* = 0.05.

### Growth and development of P. vietnamensis plantlets

**Figure 3.**
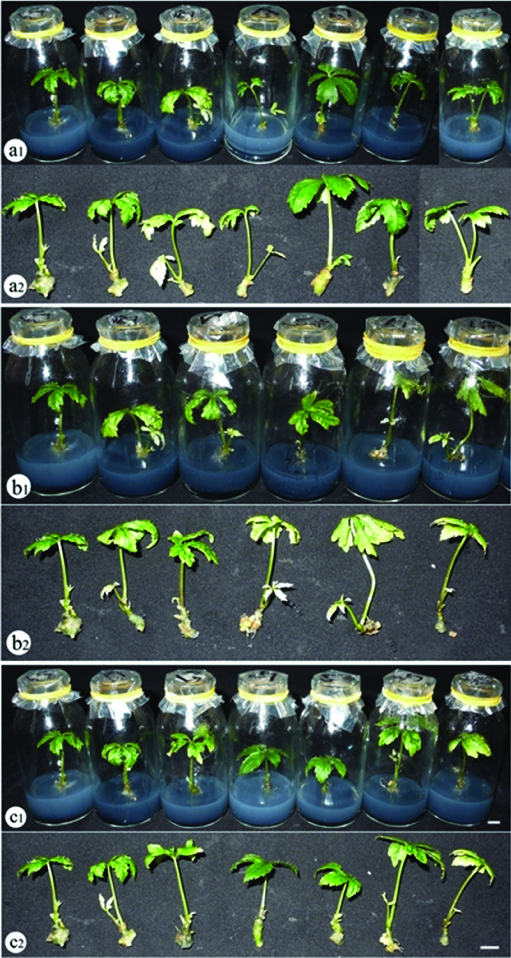
The growth and development of *P. vietnamensis* plantlets under different lighting conditions after 12 weeks of culture. a_1_, a_2_: fluorescent lamp, red, blue, green, yellow and white LEDs, and 3U compact fluorescent lamp (from left to right); b_1_, b_2_: fluorescent lamp, blue LED, red LED combined with blue LED at the ratios of 10:90, 20:80, 30:70, 40:60 and 50:50 (from left to right); c_1_, c_2_: fluorescent lamp, red LED, red LED combined with blue LED at the ratios of 90:10, 80:20, 70:30, 60:40 and 50:50 (from left to right).

Although extensive research has been carried out on the effective ratio of red LED in combination with blue LED for plant growth and development, there is no general rule in using the optimal ratio and lighting conditions for specific crops. Abdullahil Baque et al. demonstrated that the best growth of *Calanthe* plantlets was obtained under the mixture of red LED and blue LED.[15] In a study on *Cymbidium*, Tanaka et al. [16] found that the growth and development increased via the increase in photosynthesis under red LED combined with blue LED.[16] Puspa et al. [Bibr cit0016]reported that the highest plant height of grapes was observed under red LED,[17] while the best stem elongation of *Chrysanthemum* was recorded under green LED.[18] Several studies have also revealed that the combination of red LED and blue LED at the appropriate ratios enhanced the plant growth and development of *Cymbidium* (70% red LED plus 30% blue LED), *Musa* spp., *Eucalyptus*, *Spatiphyllium* and *Paphiopedilum* (80% red LED plus 20% blue LED).[19,20] It is interesting to note that in all cases of this study, the suitable lighting condition was identified. There were significant differences in plant growth and development among treatments with various lighting conditions tested (Table 3 and Figure 3). From these data, it can be seen that 60% red LED combined with 40% blue LED resulted in the highest values of fresh and dry weight, average plant height, leaf diameter and leaf length (540 mg, 82 mg, 5.4 cm, 1.62 cm and 2.90 cm, respectively), higher than those recorded under fluorescent lamp. Interestingly, SPAD index was highest when plants were cultured under 3U compact fluorescent lamp even though other parameters regarding the growth and development of *P. vietnamensis* plantlets were remarkably low under this lighting condition. Significant reduction in plant growth and development was not found with white and yellow LED, and 50% red LED plus 50% blue LED compared to fluorescent lamp. On the other hand, it is clear that green, red and blue LEDs, and the combination of red and blue LED at the ratios of 90:10, 80:20, 70:30, 40:60, 30:70, 20:80 and 10:90 resulted in a very low ability of plant growth and development.

**Table 3.** Influence of different lighting conditions on growth and development of *P. vietnamensis* plantlets after 12 weeks of culture.

Treatment	Fresh weight (mg)	Dry weight (mg)	Average height (cm)	Leaf diameter (cm)	Leaf length (cm)	SPAD
Fluorescence	453^b^	77^b^	4.7^d^	1.10^cde^	2.03^def^	24.2^e^
3U	374^f^	62^f^	4.2^g^	0.83^f^	2.20^bcd^	30.7^a^
Yellow	434^c^	62^f^	5.5^a^	1.53^ab^	3.00^a^	26.2^c^
Green	267^j^	43^j^	4.1^g^	1.10^cde^	1.73^f^	19.7^h^
White	412^d^	63^ef^	4.7^d^	1.23^cd^	2.47^bc^	26.6^b^
Red	287^i^	42^j^	3.7^h^	0.53^g^	1.83^ef^	22.0^fg^
90 red:10 blue	290^i^	42^j^	4.5^ef^	0.83^f^	2.10^def^	22.4^f^
80 red:20 blue	372^f^	63^ef^	4.5^ef^	1.07^cdef^	2.20^bcd^	25.2^d^
70 red:30 blue	387^e^	71^c^	4.8^c^	1.10^cde^	2.50^b^	25.3^d^
60 red:40 blue	540^a^	82^a^	5.4^a^	1.62^a^	2.90^a^	27.7^b^
50 red:50 blue	426^c^	70^c^	5.3^b^	1.31^bc^	2.43^bc^	23.7^e^
40 red:60 blue	390^e^	65^de^	4.9^c^	1.07^cdef^	2.20^bcd^	23.7^e^
30 red:70 blue	386^e^	55^g^	4.8^c^	1.07^cdef^	2.10^def^	21.2^g^
20 Red:80 Blue	352^g^	54^g^	4.5^ef^	1.03^def^	2.10^def^	19.7^h^
10 red:90 blue	324^h^	51^h^	4.5^ef^	0.97^ef^	2.00^def^	19.2^h^
Blue	323^h^	46^i^	4.4^f^	0.86^ef^	1.90^def^	17.5^i^

Note: Different letters (*) in the same column indicate significantly different means using Duncan's test at *P* = 0.05.

### Saponin content

In fact, light is an essential factor in the biosynthesis of secondary metabolites. Krewzaler and Hahlbrock showed that light is a major factor concerning the synthesis of flavonoid glycosides in cell culture of *Petroselinum hortense*.[21] Several studies have identified the influence of light on metabolite accumulation of *Perilla frutescens*, *Artimisia annua*, etc.[22,23] Another study which set out to determine the effect of light on the metabolic processes of ginseng (*Panax ginseng* C. A. Mayer) adventitious roots was also carried out by Park et al.[24]

**Figure 4.**
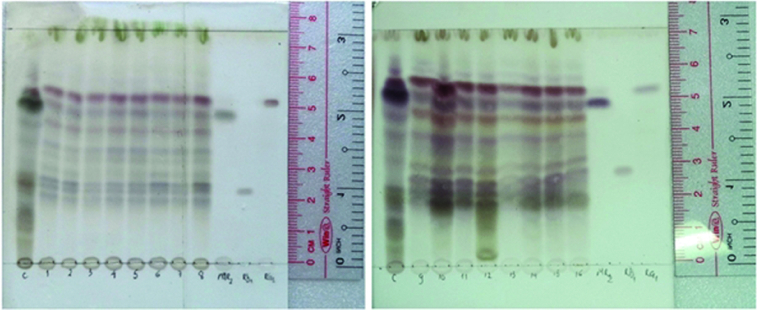
Fraction eluted from *P. vietnamensis* plantlets cultured *in vitro* samples. 1–5: red, blue, yellow, white and green LEDs; 6: fluorescent lamp; 7: 3U compact fluorescent lamp; 8–16: red LED combined with blue LED at the ratios of 90:10, 80:20, 70:30, 60:40, 50:50, 40:60, 30:70, 20:80 and 10:90, and reference samples.

However, there have been few reports on biosynthesis of secondary metabolites by *P. vietnamensis* associated with the utilisation of different light types. In this study, the correlation between lighting conditions and ginsenoside production was also tested. Thin layer chromatography was used to detect the Rg_1_, Rb_1_ and MR_2_ bands in the plantlets cultured under all the examined lighting sources (Figure 4). Moreover, similar bands of other ginsenosides of *P. vietnamensis* in the native habitat were also found in the *in vitro* samples. These results indicated that there were no significant differences between the number of ginsenosides of *in vitro P. vietnamensis* plants compared to the native ones.

**Figure 5.**
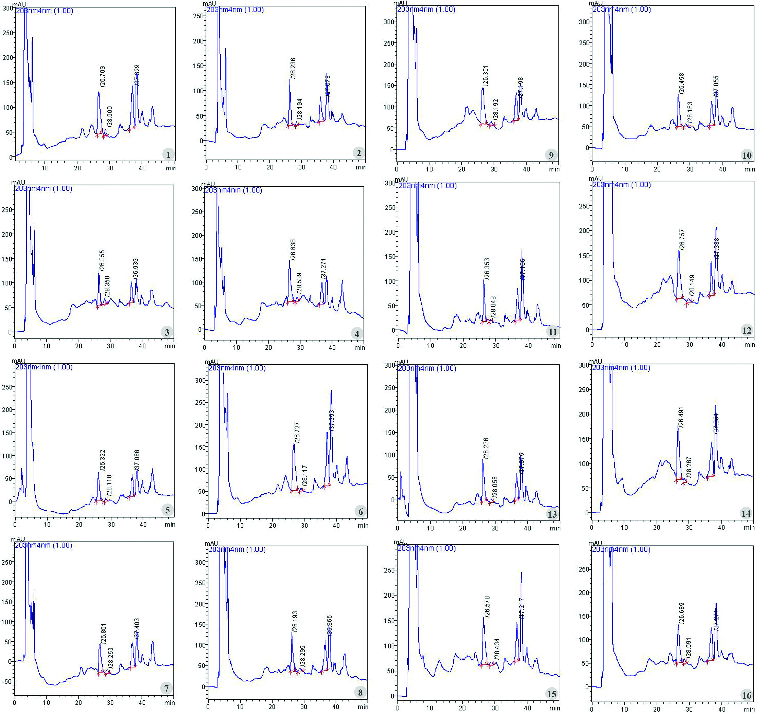
HPLC analysis of *P. vietnamensis* plantlets cultured *in vitro* with PDA detection at UV wavelength 203 nm. 1–5: red, blue, yellow, white and green LEDs; 6: fluorescent lamp, 7: 3U compact fluorescent lamp; 8–16: red LED combined with blue LED at the ratios of 90:10, 80:20, 70:30, 60:40, 50:50, 40:60, 30:70, 20:80 and 10:90.

The influence of lighting conditions on saponin accumulation of *in vitro P. vietnamensis* plants were also shown in HPLC diagram (Figure 5) and in Table 4. Rg_1_, Rb_1_ and MR_2_ were detected at the 26th, 28th and the 37th minute, respectively (Figure 5). The highest content of Rg_1_ (0.412157%) was recorded when plants maintained under fluorescent lamp, while the lowest one (0.227964%) was scored under yellow LED. The highest content of MR_2_ (0.524704%) was found under 20% red LED combined with 80% blue LED, whereas the lowest one was observed under green LED. Plants cultured under fluorescent lamps not only performed the highest Rg_1_ content but also Rb_1_ and total ginsenoside content (1.176721%) compared with those cultured under other lighting sources (Table 4).

**Table 4.** Influence of different lighting conditions on saponin accumulation of *P. vietnamensis* plantlets after 12 weeks of culture.

Treatment	Rg_1_ (%)	Rb_1_ (%)	MR_2_ (%)	Total
Fluorescence	0.412157^a^	1.176721^a^	0.307118^de^	1.895996^a^
3U	0.259674^g^	0.799265^fg^	0.124562^h^	1.183501^f^
Yellow	0.227964^h^	0.601508^j^	0.333673^c^	1.163145^f^
Green	0.328576^d^	0.904722^e^	0.054777^k^	1.288075^e^
White	0.279195^fg^	0.601960^j^	0.341122^c^	1.222277^f^
Red	0.278513^fg^	1.075454^b^	0.409299^b^	1.763266^b^
90 red:10 blue	0.283012^f^	0.994087^c^	0.313147^d^	1.590246^c^
80 red:20 blue	0.303764^e^	0.951536^d^	0.277986^f^	1.533286^cd^
70 red:30 blue	0.326776^d^	0.873868^e^	0.097099^j^	1.297743^e^
60 red:40 blue	0.335102^d^	0.867087^e^	0.113688^hi^	1.315877^e^
50 red:50 blue	0.356050^c^	0.830597^f^	0.148027^g^	1.334674^e^
40 red:60 blue	0.378348^b^	0.822521^fg^	0.298841^e^	1.499710^d^
30 red:70 blue	0.384066^b^	0.786216^g^	0.343065^c^	1.513347^d^
20 red:80 blue	0.319285^de^	0.688616^h^	0.524704^a^	1.532605^cd^
10 red:90 blue	0.278246^fg^	0.643191^i^	0.296309^e^	1.217746^f^
Blue	0.264377^fg^	0.506320^k^	0.104652^ij^	0.875349^g^

Note: Different letters (*) in the same column indicate significantly different means using Duncan's test at *P* = 0.05.

The finding from this research suggests that there is no correlation between saponin synthesis and the growth and development of *P. vietnamensis* plantlets. This relationship was found to be based on plant growth and development parameters and ginsenoside content under different lighting conditions.

## Conclusion

This study provides additional evidence for the most appropriate light type for regulation of plant growth, differentiation and metabolism. It also provides information that every developmental stage of *P. vietnamensis in vitro* requires specific lighting combination for best callus growth and plantlet development. The use of LED technology provides additional advantages in commercial tissue culture laboratories due to lower energy consumption, small size, durability, long operating lifetime, wavelength specificity, relatively cool emitting surfaces and the user's ability to determine their spectral composition. The findings suggest that the application of embryogenic callus formation technique with the suitable light combination seems to be beneficial for propagation of *P. vietnamensis*.

## Disclosure statement

No potential conflict of interest was reported by the authors.
